# Understanding the role of bats as fungal vectors in the environment

**DOI:** 10.1186/s43008-024-00161-w

**Published:** 2024-09-04

**Authors:** Xiang-Fu Liu, Samantha Chandranath Karunarathna, Saowaluck Tibpromma, K. W. Thilini Chethana, Kevin D. Hyde, Abdallah M. Elgorban, Nakarin Suwannarach, Jaturong Kumla, Peter E. Mortimer, Alice C. Hughes

**Affiliations:** 1 Center for Yunnan Plateau Biological Resources Protection and Utilization, College of Biological Resource and Food Engineering, Qujing Normal University, Qujing, 655011 Yunnan People’s Republic of China; 2 School of Biological Sciences, The University of Hong Kong, Pokfulam, 999077 Hong Kong People’s Republic of China; 3 National Institute Fundamental Studies (NIFS), Kandy, Sri Lanka; 4 School of Science, Mae Fah Luang University, Chiang Rai, 57100 Thailand; 5 Center of Excellence in Fungal Research, Mae Fah Luang University, Chiang Rai, 57100 Thailand; 6 Center of Excellence in Biotechnology Research (CEBR), King Saud University, Riyadh, Saudi Arabia; 7 Center of Excellence in Microbial Diversity and Sustainable Utilization, Chiang Mai University, Chiang Mai, 50200 Thailand; 8 Centre for Mountain Futures, Kunming Institute of Botany, Kunming, 650201 Yunnan People’s Republic of China; 9 Department of Soil Science, Stellenbosch University, Private Bag X1, Matieland South Africa

**Keywords:** Ascomycota, Basidiomycota, Bat fungi, Chiroptera, Ecology, Mucoromycota, Pathogenicity

## Abstract

Bats (Chiroptera), the second largest group of mammals, are known for their unique immune system and their ability to act as vectors for various zoonoses. Bats also act as important carriers of fungi, which include plant, animal, and human pathogens. Their roosting areas, foraging behaviors, and even migration routes make bats ideal vectors for fungi. We isolated 75 culturable fungal species from bats in Yunnan Province, China, with 36 species representing known pathogens of plants, animals, and humans, while 39 species are non-pathogenic fungi. Among these species, 77% (58 species) belonged to Ascomycota, 9% (seven species) belonged to Basidiomycota, and 13% (10 species) belonged to Mucoromycota. Even though several taxonomic studies on fungi associated with bats have been published, studies exploring the role of bats as fungal vectors are lacking. This study discusses the fungi host-specific traits and pathogenicity and the impact and ecological significance of bats as fungal vectors.

## Introduction

Bats are essential to ecosystems, pollinate and disperse seeds, and predate and control pests (Fujita and Tuttle 1991; Cleveland et al. 2006; Muscarella and Fleming 2007; Jiang et al. 2020). Bats are also known reservoirs of various zoonoses due to their unique immune systems and enhanced resilience to viral pathogens, some of which have been directly linked to human spillovers and epidemics (SARS, COVID-19, Hendra, Nipah, MERS). Regardless of their unique resilience to viruses, fungal pathogens (e.g., *Pseudogymnoascus destructans*) have been linked to the deaths of over seven million bats in the US alone (Cheng et al. 2021). Although more than 400 fungi species associated with bats have been reported in previous studies and showed a high diversity, the fungal interactions with bats are much less known (Johnson et al. 2013; Vanderwolf et al. 2013; Kokurewicz et al. 2016; Holz et al. 2018; Cunha et al. 2020; Ogórek et al. 2020; Liu et al. 2023). With the discoveries of pathogenic fungi on bats in China (Karunarathna et al. 2020), understanding interactions between bats and fungi, and their role as fungal vectors across landscapes is critically important (Karunarathna et al. 2023; Liu et al. 2023). Fungal pathogens are particularly complex in that some fungi can show different growth modes, enabling them to be both benign in one mode and plant and animal pathogenic in another, emphasizing the importance of understanding how fungi distribute across landscapes.

Three main factors contribute to bats being ideal vectors for fungal pathogens. Bat hibernacula and roosts such as caves and mines are optimal environments for fungal growth, featuring stable, mild temperatures, high humidity, and rich sources of organic matter (Held et al. 2020). In addition, bats constantly fly travel between caves, forests, croplands, and human settlements during their daily and seasonal activities (Thomas and Jung 2019). Thus, fungal pathogens can be easily transferred between land use types. Feeding behaviors and migratory routes of bats bring them into contact with a range of fungi, including numerous plant pathogens, indirectly threatening human health, and all the bats in this study were caught in close proximity to certain crops, including bananas, rice, and a range of fruit trees.

In the context of understanding these interactions, it is important to understand both bats' capacity to carry viable fungal pathogens and how bats interact with landscapes and, therefore, determine their viability to spread fungi. Most bat species (except large-bodied pteropids) roost in enclosed environments during the day, often in groups of thousands or even millions of individuals. There are 678 known bat species (48%) that occupy caves (Tanalgo et al. 2022), in addition to the species that live in groups in human structures and tree hollows. Some fungal pathogens (such as chytrid fungi in amphibians—*Batrachochytrium dendrobatidis*) survive poorly when exposed to direct sunlight (Longcore et al. 1999). Thus, bats, as species that have limited exposure to the sun and frequently occupy thermally stable, moist environments, provide an ideal medium for fungal growth and strengthen their capacity to spread fungal pathogens rapidly, as well as host various non-pathogenic fungi. Based on this, we expect cave-roosting bats, especially those that roost in close proximity and in large numbers, to harbour more fungi. Conversely, species that roost in tree hollows (which typically live in smaller groups) may host fewer species of fungi but are likely to show greater similarities in the fungal composition on different individuals, as they are more likely to be the same species and are forced into direct contact, but there is limited air-circulation in small tree cavities as they are crowded and often have a single entrance so the spread of fungi would likely be via contact (Willis and Brigham 2007).

The other factor responsible for exposure to how bats use space is based on species-specific traits on adaptions to habitat openness and diet. Bat ecomorphology links precisely to the degree of clutter in the environment, with species traits such as echolocation call and wing morphology directly related to habitat type. Species adapted to highly cluttered forest environments may be able to use tree plantations but are unlikely to traverse crop fields, whereas species adapted to open environments may forage over crops. Species that forage in these landscapes will have the capacity to act as vectors between roosting and foraging environments and thus spread fungal pathogens across landscapes. Furthermore, bats consume various disease vectors and thus have the capacity to reduce various vector-borne diseases (Puig‐Montserrat et al. 2020). Understanding where these risks exist is an important first step to understanding how such threats of transfer of plant and animal pathogens can be mitigated between natural and human environments. It is also important to note that chemicals sprayed on crops are likely to control the natural ability of many species to respond immunologically to various pathogens, and in the case of fungi, the switch that enables them to become pathogenic in immunocompromised hosts is poorly known (Lionakis et al. 2023). However, it should be noted that for fungi to be identified to the species level and described, they must first be cultured, meaning that a host of fungal species that cannot be cultured in lab conditions is likely to be overlooked.

Here, using data from bats across Yunnan Province, China, we investigate their role as vectors of various fungal pathogens. We also explore the influence of species-specific traits such as roosting habits and colony size and how fungal pathogens on different individuals relate to the degree of environmental modification in the environment sampled. Following this, we discuss the implications of these interactions and how such risks could be mitigated through targeted interventions, finally identifying major knowledge gaps that require further study.

## Methods

### Bat research methods-survey protocol, site selection, species identification

Most bats were captured and sampled during weekly survey work inside the two forest patches in the Xishuangbanna Tropical Botanical Garden (XTBG) and were permitted by the Xishuangbanna National Nature Reserve and Xishuangbanna Tropical Botanical Garden. Surveys were made by setting three harp traps with four banks each. Harp traps were set to be ready for 30 min prior to sunset and closed at around 10.30 pm when capture rates dropped. However, additional sampling sites included caves at several sites near Kunming (Table 1). Bats were then collected into cloth bags and taken to a central area for processing, after which they were released.

**Table 1:** Overall diversity of fungi and bat species diversity per site

Location	Bat_specs	Bat_inds	Fungi_specs	Mean fungi species per bat
Xishan district, long cave	4	7	4	1.00
Rainforest in XTBG	6	49	27	4.50
Yuxi, Yimen County, Pubei Village	9	50	19	2.11
Limestone forest in XTBG	11	66	38	3.45

Bat_specs: Bat species; Bat_inds: Bat individuals; Fungi_specs: Fungal species

Limestone forest and rainforest areas are found within Xishuangbanna Tropical Botanical Garden, whilst the limestone forest (and associated cave) did have cropland on one side. Pubeixiang cave, which has a river running through it and thus has a higher than normal humidity, was nestled in an agricultural landscape, with crops and livestock frequenting this area. In addition, rodents were also recorded during visits to the site. Yimen cave is smaller in size, found in the same general area, and disturbed, with a smaller bat population and fewer species than the Pubeixiang cave site.

Standard morphological measurements of the captured bat species were made using digital calipers (Mitutoyo Absolute Series-500, with an accuracy of 0.01 mm) and included forearm, head, body, hindfoot, tibia, ear length, and nose leaf width and length, and photographs of the wing taken on gridded paper to calculate flight performance metrics (wing area, aspect ratio, wing-loading, and wingtip angle). Bat calls were recorded using a Pettersson M500-384 (Pettersson Elektronik AB; www.batsound.com) and later analyzed in BatSound ver4 (Pettersson electronic AB, Uppsala Sweden) at a sampling rate of 44.1 kHz and spectrograms were set at 1,024 sampling site FFT. Each individual bat was photographed using a FUJIFILM X100F camera (https://fujifilm-x.com/global/products/cameras/x100f/), including photos of the front of the face, the profile from the side, and the wing, which were used to validate species identity. Species identification was based on measures from Francis (2019), and confirmed by Cytochrome c oxidase subunit I (CO1) barcoding of bats at each locality based on tissue samples taken with a 1.8 mm biopsy punch taken from the wing and stored in 99% ethanol for later processing at the Southern China DNA barcoding centre, full details are noted in Chornelia et al. (2022), and all work was conducted with permission from Xishuangbanna Tropical Botanical Garden, and Xishuangbanna Nature Reserve. Samples were taken from live bats and released at the end of each evening, once all bats had been processed.

### Sampling methods for taking fungal swabs

Samples of fungi were collected using sterile swabs that were pre-moistened with sterilized water plus chloramphenicol *(*0.1 mg/L*)*. These were gently rolled back and forth three times across the bat fur, the wing membrane, and the feet (Liu et al. 2023). Swabs were then individually placed in sterilized 50 mL centrifuge tubes containing 15 mL sterilised water plus chloramphenicol (0.1 mg/L), labelled, and stored at 4 ℃ until the samples were cultured (Cunha et al. 2020; Liu et al. 2023).

### Fungal culture

At the laboratory, the conical centrifuge tubes containing the swabs were shaken, then, using a sterilized cotton bud, the suspension was spread on potato dextrose agar (PDA, Oxoid, England) plates containing amoxicillin (50 μg/mL), and the procedure was repeated in triplicate. The PDA plates were incubated at room temperature (20–25 °C) until individual fungal colonies were visible. These individual fungal colonies were then sub-cultured on new PDA plates in triplicate and incubated at room temperature. All fungal strains were stored at 4 °C for further studies. Full isolates and taxonomic identification were based on morphology and multigene phylogeny; the details are provided in Liu et al. (2023).

### Species assessment and data analysis

The tables and charts used for species assessment and data analysis were created using in Microsoft Excel 2019. Venn diagrams were made on website E Venn (Yang et al. 2024; https://www.ehbio.com/test/venn/#/) or eulerr (https://eulerr.co/) and annotated in Microsoft PowerPoint 2019.

## Results

### The overview of bats and fungi

In total, 164 bats belonging to 19 species were included in our study, of which 74 bats were sampled for just wing fungi, and 90 were sampled for fungi from the wings, legs, and body. A total of 68 bats were found to have culturable fungi, whereas 96 had none, and cultured fungi included 75 different species, with 48% (36 species) representing known pathogens of plants, animals, humans, mushrooms and insects, and 52% (39 species) representing known non-pathogenic fungi (Liu et al. 2023) (Table 2). Incidence varied by site and species, with a single bat species hosting as many as nine species of fungi. Within bat species, each individual hosted different fungal profile. Whilst some fungi were more prevalent in certain groups, no bat species with more than two individuals universally hosted the same fungi, and relatively few showed evidence of host-specificity.

**Table 2:** Culturable fungal species isolated from bats and their pathogenicity and instances. NA indicated no available information

Species	Disease(s) caused	Isolated bat species	Original code	Microhabitat	Bat feeding habit
**Ascomycota**					
*Amphichorda yunnanensis*	NA	*Rhinolophus affinis*	YM-24-W1	Wings	Insects
		*Rhinolophus affinis*	YM-24-W4	Wings	Insects
		*Rhinolophus siamensis*	YM-18-W6	Wings	Insects
*Apiospora arundinis*	Onychomycosis—human pathogen (Dylag et al. 2017; Vettorato et al. 2020); plant pathogen (leaf edge spot of peach, leaf blight of tea, wet root rot of *Pseudostellaria* heterophylla) (Thangaraj et al. 2019; Ji et al. 2020; Xiao et al.^2024^)	*Miniopterus schreibersii*	XS-5-L2	Legs	Insects
		*Myotis pilosus*	XS-142-L	Legs	Fish
*Apiospora marii*	Plant pathogen (wilt, dieback and tree decline of olive) (Gerin et al. 2020; Farr and Rossman 2022)	*Rhinolophus sinicus*	YM-56-W4	Wings	Insects
		*Rhinolophus sinicus*	YM-56-W4-2	Wings	Insects
*Apiospora vietnamensis*	NA	*Hipposideros pomona*	YM-66-B4	Body	Insects
		*Hipposideros pomona*	YM-66-B4-2	Body	Insects
*Apiospora xishuangbannaensis*	NA	*Rhinolophus pusillus*	25	Wings	Insects
		*Rhinolophus pusillus*	25-B	Wings	Insects
*Aspergillus candidus*	Causes respiratory disease and onychomycosis in humans (Krysinska-Traczyk and Dutkiewicz 2000; Ahmadi et al. 2012)	*Hipposideros armiger*	XS-1-B2	Body	Insects
		*Hipposideros armiger*	XS-1-B2-2	Body	Insects
*Aspergillus creber*	Causes fungal infections in immunosuppressed individuals (Siqueira et al. 2016)	*Rhinolophus affinis*	YM-24-W6	Wings	Insects
		*Rhinolophus affinis*	YM-24-W6-2	Wings	Insects
*Aureobasidium xishuangbannaensis*	NA	*Myotis laniger*	60-E	Wings	Insects
		*Myotis laniger*	60-D	Wings	Insects
		*Rhinolophus malayanus*	28-A	Wings	Insects
*Candida glabrata*	Human pathogenic on immunocompromised hosts (mucosal tissue infection and candidal arthritison) (Fidel et al. 1999; Hassan et al. 2021)	*Hipposideros larvatus*	69	Wings	Insects
		*Kerivoula papillosa*	74-A	Wings	Insects
*Candida orthopsilosis*	Causes fungal keratitis, fungemias and septic arthritis on humans (Blanco-Blanco et al. 2014; Heslop et al. 2015)	*Rhinolophus malayanus*	55-B	Wings	Insects
*Candida parapsilosis*	Causes candidiasis in humans (Trofa et al. 2008)	*Rhinolophus stheno*	56-B	Wings	Insects
		*Rhinolophus stheno*	56-G	Wings	Insects
		*Rhinolophus stheno*	XTBG-2-B1	Body	Insects
		*Rhinolophus stheno*	XTBG-2-B2	Body	Insects
*Candida saopaulonensis*	Human pathogen (fungi infections in premature infant with sepsis) (Ning et al. 2024)	*Hipposideros pomona*	43	Wings	Insects
		*Hipposideros pomona*	43-A	Wings	Insects
		*Hipposideros pomona*	43-A1	Wings	Insects
*Chaetomium anastomosans*	Plant pathogen (diseased root of *Saccharum officinarum*) (Raza et al. 2019); Human pathogen (eye infections) (Walther et al. 2021)	*Myotis muricola*	35	Wings	Insects and small invertebrates
*Chaetomium globosum*	Mycotoxin producing species, mycotoxins can be lethal to mammalian cells (Fogle et al. 2008)	*Rhinolophus malayanus*	XTBG-6-W6	Wings	Insects
		*Rhinolophus malayanus*	XTBG-6-W6-B	Wings	Insects
*Clonostachys pityrodes*	Mycoparasitic fungus (Bich et al. 2021)	*Rhinolophus malayanus*	63-D	Wings	Insects
*Clonostachys rhinolophicola*	NA	*Rhinolophus stheno*	56-F	Wings	Insects
		*Hipposideros larvatus*	62	Wings	Insects
*Coniochaeta* sp.	NA	*Rhinolophus malayanus*	57-A	Wings	Insects
		*Rhinolophus malayanus*	57-D	Wings	Insects
*Daldinia eschscholtzii*	Human pathogen (fungal infection) (Ng et al. 2016)	*Rhinolophus malayanus*	XTBG-6-W7	Wings	Insects
*Debaryomyces vindobonensis*	Fungal infection in bats (Tamayo et al. 2021)	*Rhinolophus malayanus*	XTBG-6-W8	Wings	Insects
		*Rhinolophus malayanus*	XTBG-6-W8-B	Wings	Insects
*Fusarium annulatum*	Plant pathogen (*Fusarium* rot of cantaloupe melons) (Parra et al. 2022)	*Hipposideros pomona*	43-D	Wings	Insects
		*Hipposideros pomona*	43-D2	Wings	Insects
*Fusarium hipposidericola*	NA	*Rhinolophus malayanus*	36-D1	Wings	Insects
		*Rhinolophus malayanus*	65-C	Wings	Insects
		*Rhinolophus malayanus*	65-D	Wings	Insects
		*Rhinolophus stheno*	40-C	Wings	Insects
		*Rhinolophus stheno*	40-E	Wings	Insects
*Fusarium luffae*	Plant pathogen (leaf blight on loquat, pokkah boeng of maize) (Parime et al. 2022; Zhang et al. 2023a, b, c)	*Rhinolophus malayanus*	49-A	Wings	Insects
*Fusarium menglaense*	NA	*Rhinolophus malayanus*	39	Wings	Insects
		*Rhinolophus malayanus*	39-B	Wings	Insects
*Fusarium rhinolophicola*	NA	*Rhinolophus malayanus*	38	Wings	Insects
		*Rhinolophus malayanus*	38-B	Wings	Insects
		*Rhinolophus malayanus*	38-C	Wings	Insects
*Fusarium* sp.	NA	*Hipposideros pomona*	64-A	Wings	Insects
		*Hipposideros pomona*	64-B	Wings	Insects
		*Rhinolophus malayanus*	28-C	Wings	Insects
		*Rhinolophus malayanus*	33	Wings	Insects
		*Rhinolophus malayanus*	33-C	Wings	Insects
*Fusarium xishuangbannaense*	NA	*Rhinolophus malayanus*	55-A	Wings	Insects
		*Rhinolophus malayanus*	55-D	Wings	Insects
*Fusarium yunnanense*	NA	*Rhinolophus malayanus*	39-A	Wings	Insects
		*Rhinolophus malayanus*	39-C	Wings	Insects
*Hyphopichia burtonii*	Cutaneous mycosis in barbastelle bat (Simpson et al. 2013); Human pathogen (fungal peritonitis) (Chamroensakchai et al. 2021)	*Hipposideros larvatus*	69-A	Wings	Insects
		*Rhinolophus malayanus*	24C	Wings	Insects
		*Rhinolophus malayanus*	28-B	Wings	Insects
*Hyphopichia lachancei*	NA	*Hipposideros pomona*	54-A	Wings	Insects
*Hypoxylon investiens*	Plant pathogen (*Hypoxylon* wood rot in tea) (Grand 1985; Otieno 1993)	*Hipposideros larvatus*	37	Wings	Insects
		*Hipposideros larvatus*	37-B	Wings	Insects
*Hypoxylon monticulosum*	NA	*Rhinolophus malayanus*	XTBG-6-W5	Wings	Insects
		*Rhinolophus malayanus*	XTBG-6-W5-B	Wings	Insects
*Metschnikowia* sp.	NA	*Miniopterus schreibersii*	XS-5-W	Wings	Insects
		*Miniopterus schreibersii*	XS-5-W2	Wings	Insects
*Metschnikowia koreensis*	NA	*Hipposideros larvatus*	37-D	Wings	Insects
*Meyerozyma carpophila*	NA	*Rhinolophus rex*	YM-16-W4	Wings	Insects
		*Rhinolophus siamensis*	YM-45-W	Wings	Insects
*Meyerozyma guilliermondii*	Human pathogenic on immunocompromised hosts (Lim et al. 2023)	*Rhinolophus stheno*	31-B	Wings	Insects
		*Hipposideros larvatus*	62-A	Wings	Insects
Montagnula sp.	NA	*Hipposideros larvatus*	62-B	Wings	Insects
*Neopestalotiopsis paeoniae-suffruticosae*	Pathogenic on diseased branches of *Paeonia suffruticosa* (Li et al. 2022)	*Rhinolophus stheno*	70	Wings	Insects
		*Rhinolophus stheno*	70-B	Wings	Insects
*Neopestalotiopsis xishuangbannaensis*	NA	*Kerivoula hardwickii*	45	Wings	Insects
		*Kerivoula hardwickii*	45-B	Wings	Insects
*Parasarocladium gamsii*	NA	*Rhinolophus malayanus*	57-C	Wings	Insects
		*Myotis laniger*	68-C	Wings	Insects
*Penicillium brevicompactum*	Weak pathogen on fruits; mycoparasitic (blue mold disease of *Grifola frondosa*) (Tian et al. 2017); human pathogen (invasive pulmonary mycosis) (De La Cámaraet al. 1996)	*Rhinolophus affinis*	YM-24-W3	Wings	Insects
		*Rhinolophus rex*	YM-17-B2	Body	Insects
		*Rhinolophus rex*	YM-30-L	Legs	Insects
		*Rhinolophus rex*	YM-30-W2	Wings	Insects
		*Rhinolophus siamensis*	YM-18-L	Legs	Insects
		*Rhinolophus siamensis*	YM-45-L	Legs	Insects
		*Rhinolophus siamensis*	YM-45-W1	Wings	Insects
		*Rhinolophus sinicus*	YM-52-B1	Body	Insects
*Penicillium coprophilum*	Insect pathogen (a pathogen on mosquitoes) (Costa et al. 1998)	*Rhinolophus sinicus*	YM-56-W6	Wings	Insects
		*Rhinolophus sinicus*	YM-56-W3	Wings	Insects
*Penicillium glabrum*	Plant pathogen (infecting on strawberries, rot of pomegranate) (Spadaro et al. 2010; Barreto et al. 2011)	*Rhinolophus sinicus*	YM-56-W1	Wings	Insects
		*Rhinolophus sinicus*	YM-56-W7	Wings	Insects
*Pestalotiopsis trachicarpicola*	Plant pathogen (leaf spots on *Gentiana rhodantha, Trachycarpus fortunei*, and twig blight of *Pinus bungeana*) (Qi et al. 2021; Zhang et al. 2012, 2021)	*Rhinolophus pusillus*	XS-31-W2	Wings	Insects
*Phialemoniopsis hipposidericola*	NA	*Hipposideros larvatus*	62-D	Wings	Insects
		*Hipposideros larvatus*	62-D2	Wings	Insects
*Phialemoniopsis xishuangbannaensis*	NA	*Hipposideros larvatus*	62-C	Wings	Insects
		*Hipposideros larvatus*	62-E	Wings	Insects
		*Hipposideros larvatus*	62-G	Wings	Insects
		*Hipposideros larvatus*	62-G1	Wings	Insects
*Saccharomyces cerevisiae*	An opportunistic human pathogen, though of relatively low virulence (Murphy and Kavanagh 1999; Goldstein and McCusker 2001)	*Rhinolophus affinis*	YM-105-W2	Wings	Insects
		*Rhinolophus malayanus*	63-A	Wings	Insects
		*Rhinolophus stheno*	40-A	Wings	Insects
*Saccharomycopsis crataegensis*	NA	*Rhinolophus malayanus*	34-A	Wings	Insects
		*Rhinolophus malayanus*	34-B	Wings	Insects
*Saccharomycopsis fibuligera*	NA	*Rhinolophus stheno*	56-E	Wings	Insects
		*Rhinolophus malayanus*	63-C	Wings	Insects
*Sarocladium zeae*	NA	*Rhinolophus stheno*	56-A	Wings	Insects
		*Rhinolophus stheno*	56-B	Wings	Insects
*Schwanniomyces polymorphus*	NA	*Rhinolophus sinicus*	YM-52-B2	Body	Insects
		*Rhinolophus sinicus*	YM-52-B2-2	Legs	Insects
*Scopulariopsis brevicaulis*	Human pathogen (Cuenca-Estrella et al. 2003; Woudenberg et al. 2017)	*Rhinolophus siamensis*	YM-18-W2	Legs	Insects
		*Rhinolophus siamensis*	YM-18-W2-2	Wings	Insects
		*Rhinolophus siamensis*	YM-18-W5	Wings	Insects
		*Rhinolophus siamensis*	YM-18-W5-2	Wings	Insects
*Trichoderma hipposiderocola*	NA	*Hipposideros pomona*	YM-66-B2	Body	Insects
		*Hipposideros pomona*	YM-66-L1	Legs	Insects
		*Miniopterus schreibersii*	YM-62-L1	Legs	Insects
		*Rhinolophus affinis*	YM-88-B1	Body	Insects
		*Rhinolophus affinis*	YM-88-L2	Legs	Insects
*Trichoderma inconspicuum*	NA	*Miniopterus schreibersii*	YM-62-B2	Body	Insects
*Trichoderma obovatum*	NA	*Hipposideros pomona*	YM-66-B3	Body	Insects
		*Hipposideros pomona*	YM-66-B5	Body	Insects
		*Hipposideros pomona*	YM-66-B6	Body	Insects
		*Miniopterus schreibersii*	YM-62-B2	Body	Insects
		*Rhinolophus rex*	YM-16-B	Body	Insects
*Trichoderma rhinolophicola*	NA	*Rhinolophus malayanus*	55-F	Wings	Insects
		*Rhinolophus malayanus*	55-F2	Wings	Insects
*Trichoderma xishuangbannaense*	NA	*Rhinolophus malayanus*	73-D	Wings	Insects
		*Rhinolophus malayanus*	73-D2	Wings	Insects
*Xylaria adscendens*	NA	*Kerivoula papillosa*	74-C	Wings	Insects
		*Kerivoula papillosa*	74-C2	Wings	Insects
*Xylaria curta*	NA	*Rhinolophus malayanus*	30-D	Wings	Insects
		*Rhinolophus malayanus*	30-D2	Wings	Insects
**Basidiomycota**					
*Ceriporia lacerata*	White rot causing fungus (Suhara et al. 2003; Sui and Yuan 2023)	*Hipposideros larvatus*	37-E	Wings	Insects
*Coprinopsis minuta*	NA	*Rhinolophus malayanus*	30-B	Wings	Insects
		*Rhinolophus malayanus*	30-B2	Wings	Insects
*Cutaneotrichosporon dermatis*	Human pathogenic on immunocompromised hosts (Yoo et al. 2022)	*Hipposideros larvatus*	62-A1	Wings	Insects
		*Hipposideros pomona*	54-B	Wings	Insects
		*Rhinolophus affinis*	YM-24-B2	Body	Insects
		*Rhinolophus affinis*	YM-24-W5	Wings	Insects
		*Rhinolophus rex*	YM-16-B1	Body	Insects
*Phlebia acerina*	White-rot (Kumar et al. 2018; Zhang et al. 2023a, b, c)	*Myotis laniger*	60-C	Wings	Insects
		*Myotis laniger*	60-C2	Wings	Insects
*Phlebia floridensis*	White-rot (Magaña-Ortiz et al. 2024)	*Hipposideros pomona*	43-B	Wings	Insects
		*Hipposideros pomona*	43-B2	Wings	Insects
*Psathyrella candolleana*	NA	*Rhinolophus sinicus*	73-E	Wings	Insects
		*Rhinolophus sinicus*	74-E-2	Wings	Insects
*Rhodotorula mucilaginosa*	Human pathogen (onychomycosis) (Larone 1995; Wirth and Goldani 2012)	*Myotis laniger*	60-A	Wings	Insects
		*Myotis laniger*	68-A	Wings	Insects
		*Rhinolophus affinis*	YM-24-B	Body	Insects
		*Rhinolophus malayanus*	29-A	Wings	Insects
		*Rhinolophus malayanus*	41-A	Wings	Insects
		*Rhinolophus malayanus*	55-C	Wings	Insects
		*Rhinolophus malayanus*	63-B	Wings	Insects
		*Rhinolophus malayanus*	65-A	Wings	Insects
		*Rhinolophus siamensis*	26	Wings	Insects
		*Rhinolophus stheno*	31-C	Wings	Insects
		*Rhinolophus stheno*	40-B	Wings	Insects
		*Rhinolophus stheno*	56	Wings	Insects
**Mucoromycota**					
*Mucor breviphorus*	NA	*Rhinolophus malayanus*	44-A	Wings	Insects
*Mucor changshaensis*	NA	*Rhinolophus malayanus*	72	Wings	Insects
*Mucor circinelloides*	Cutaneous infections of humans (de Hoog et al. 2000; Samson et al. 2000; Vellanki et al. 2018); infect animals (cattle, swine, fowl, and platypus) (Rippon 1988; Pitt and Hocking 1999)	*Rhinolophus malayanus*	44-B	Wings	Insects
*Mucor ellipsoideus*	Human pathogen (chronic renal failure) (Gupta et al. 1989; Alvarez et al. 2011; Prakash and Chakrabarti 2019)	*Hipposideros larvatus*	71	Wings	Insects
		*Hipposideros larvatus*	71-A	Wings	Insects
		*Rhinolophus malayanus*	33-B	Wings	Insects
*Mucor irregularis*	Human pathogen (an emerging fungal pathogen that cause cutaneous infection of humans and could cause death; rhinofacial mucormycosis) (Hemashettar et al. 2011; Chander et al. 2015); Fungicolous on Pleurotus sp. (Rammaert et al. 2014; Jayasiri et al. 2015)	*Rhinolophus malayanus*	44-A1	Wings	Insects
		*Rhinolophus stheno*	67-B	Wings	Insects
*Mucor plumbeus*	Able to elicit an immune response in humans by activating the complement system (Domsch et al. 1995; Kirk 1997; Granja et al. 2010; Wagner et al. 2020; Boraschi et al. 2020)	*Rhinolophus rex*	YM-30-W1	Wings	Insects
*Mucor pseudolusitanicus*	NA	*Miniopterus schreibersii*	XS-22-W	Wings	Insects
*Mucor racemosus*	Opportunistic pathogen of immunocompromised individuals such as children, elderly and diseased patients (Sarbhoy 1966; Inderlied et al. 1985; Alvarez et al. 2011; Gidalishova et al. 2023)	*Rhinolophus affinis*	YM-105-W1	Wings	Insects
*Mucor* sp.	NA	*Rhinolophus malayanus*	42	Wings	Insects
		*Rhinolophus malayanus*	42-B	Wings	Insects
*Mucor variicolumellatus*	Human pathogen (infection of human) Walther (Wagner et al. 2020)	*Rhinolophus malayanus*	33-A	Wings	Insects
		*Rhinolophus rex*	YM-17-W2	Wings	Insects
		*Rhinolophus rex*	YM-17-W3	Wings	Insects
		*Rhinolophus siamensis*	27	Wings	Insects

Interestingly, when we collected samples from different bat body parts, fungi could sometimes be cultured in high numbers from some body parts whilst being completely absent from others (Figs. 2 and 3). For example, two individuals had no fungi on their bodies or legs but had 4–5 species of fungi on their wings, and three individuals only had fungi on their bodies but only one species on each (Table 3). Additionally, one species had different fungi on the legs and wing but none on the body, and one had the same fungi on the body and legs but none on the wing. Thus, wings are likely to host the most diverse community of fungi, whereas other body parts may share fungal species and host a lower diversity of fungi (Figs. 2 and S1). Furthermore, more cultures and species were found on the wings than other body parts; for example, fungi were only isolated from the wings of *Rhinolophus malayanus* and *R. sinicus*, despite multiple body parts being sampled. In contrast, fungi found on other body parts of *R. siamensis* were also found on the wings (Fig S1, Table 2). In *R. stheno*, fungi isolated from the body of some individuals were found on the wings of other individuals. Even though the majority of fungi are found on the wings of *R. rex* and *R. affinis*, *Trichoderma hipposiderocola* was found on the bodies and legs of the species, and *Penicillium brevicompactum* was found on the wings, legs, and body of several *Rhinolophus* species. In *R. sinicus*, different individuals had multiple species either in the wing, body, or leg. Rhinolophids exhibited a majority of fungi on their wings; for example, of individuals sampled from their wings, body, and legs, 51 of the 59 fungal cultures were only from the wings (Fig. 2; Table S1), two from both the wings and body, and one species shared among all three. Thus, for most Rhinolophids (with the exception of *R. rex* and possibly *R. stheno*), wings were the most important location for hosting fungi.

**Fig. 1:**
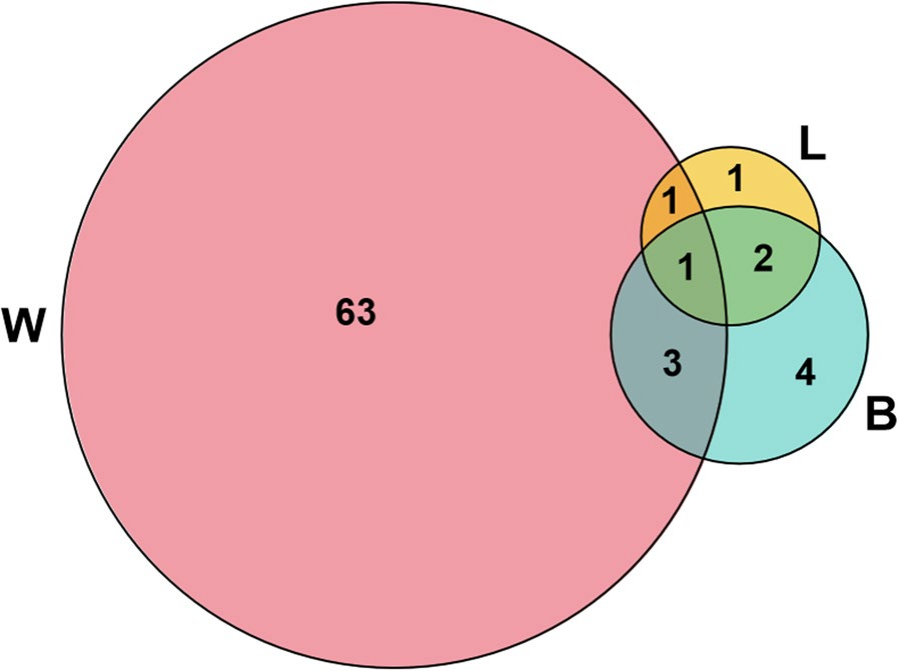
Numbers of fungal species cultured from each body part of bats (W-wing, B-body, L-legs)

**Fig. 2:**
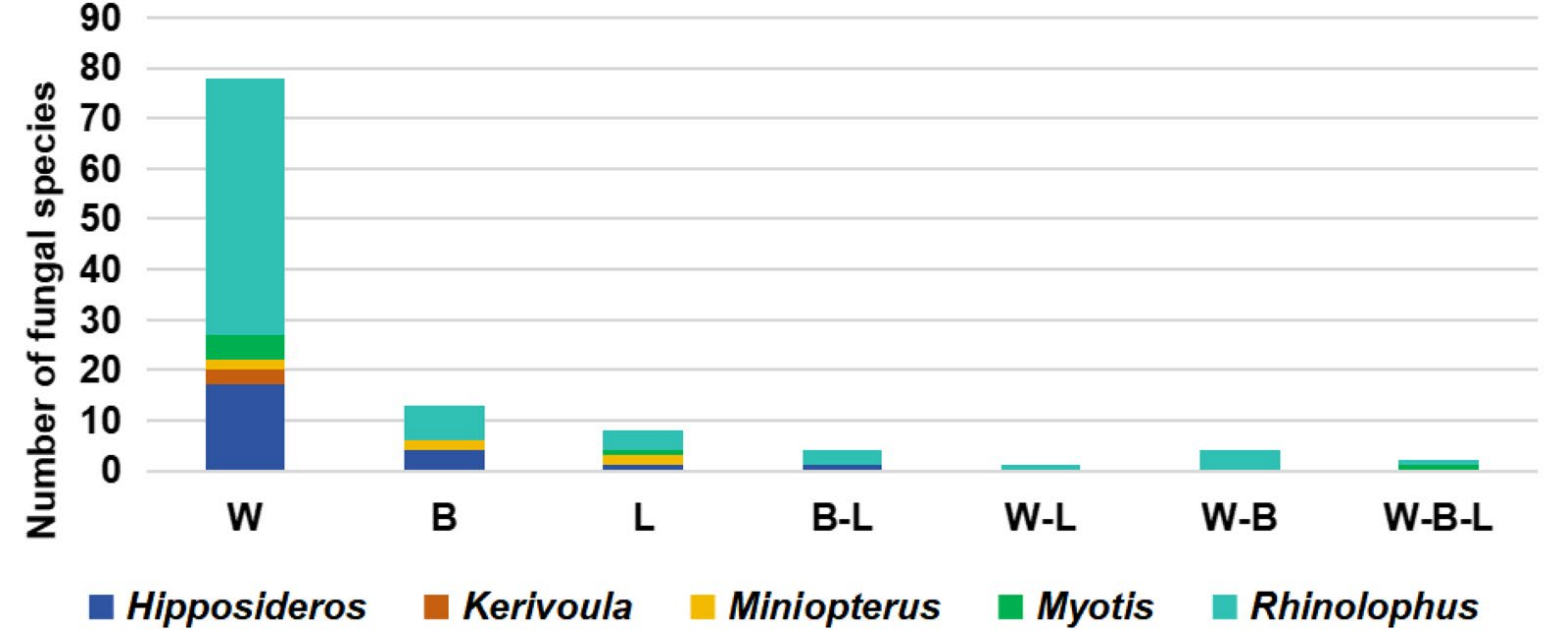
Numbers of fungal species cultured from each body part (W-wing, B-body, L-legs) of the individual bats from each group (*Hipposideros*, *Kerivoula*, *Miniopterus*, *Myotis*, and *Rhinolophus*) when samples were collected from all body parts

**Table 3:** Bat species traits and infection rates

Species	Colony size	Distance from other individuals	Roost type	% bats with fungi	Nbats total	Fungal incidences	Fungal species	Fungal genera	av_fungi_bat (IN)	av_fungi_bat (T)
*Aselliscus stoliczkanus*	Medium	Large	Cave	0	1	0	0	0		
*Hipposideros armiger*	Medium	Large	Cave	25	4	2	1	1	0.25	1
*Hipposideros cineraceus*	Small	Large	Cave	0	1	0	0	0		
*Hipposideros larvatus*	Medium	Large	Cave	36.36	11	18	12	9	3	1.09
*Hipposideros pomona*	Medium	Small	Cave	57.14	7	18	11	7	2.75	1.57
*Kerivoula papillosa*	Small/solitary	Large	Tree	100	1	3	2	2	2	2
*Miniopterus schreibersii*	Small	Small	Cave	75	4	7	6	4	2	1.5
*Myotis laniger*	Small	Small	Flexible	40	5	7	4	4	2	0.8
*Myotis muricola*	Small	Small	Flexible	100	1	1	1	1	1	1
*Myotis pilosus*	Small	Small	Flexible	100	1	1	1	1	1	1
*Rhinolophus affinis*	Large	Small	Cave	100	3	13	8	8	2.67	2.67
*Rhinolophus malayanus*	Large	Small	Cave	64.52	31	57	33	19	1.65	1.06
*Rhinolophus pusillus*	Large	Small	Cave	100	2	3	2	2	1	1
*Rhinolophus rex*	Small	Large	Cave	100	3	9	6	5	2	2
*Rhinolophus siamensis*	Small	Small	Cave	80	5	11	6	5	1.5	1.2
*Rhinolophus sinicus*	Medium	Small	Cave	80	5	11	6	4	1.5	1.2
*Rhinolophus stheno*	Medium	Small	Cave	41.18	17	18	10	10	1.43	0.59

Note, for some species, such as *R. rex*, many fungal species were exclusive to the body and were not found on wings, indicating that fur type and length may influence the capacity to act as a fungal vector. IN indicated the average number of fungi per bat out of all bats with fungi; T indicated the average number of fungi per bat out of all bats. Colony size into either: small (10 s of individuals), medium (tens-hundreds of individuals), large (hundreds to thousands of individuals). Distance from other individuals into either: small medium, large)

However, other bat species show different patterns, exhibiting a high fungal prevalence in the body and legs in Hipposiderids and *Myotis* and none exclusive to wings or shared between body parts. These patterns are likely to relate to roosting habits and colony size of bats (though it may relate to morphological differences Cheney et al. 2017), but wings typically hosted a much greater diversity of fungi (> 5) than body and legs, which hosted a maximum of around two fungal species on an individual.

**Fig. 3:**
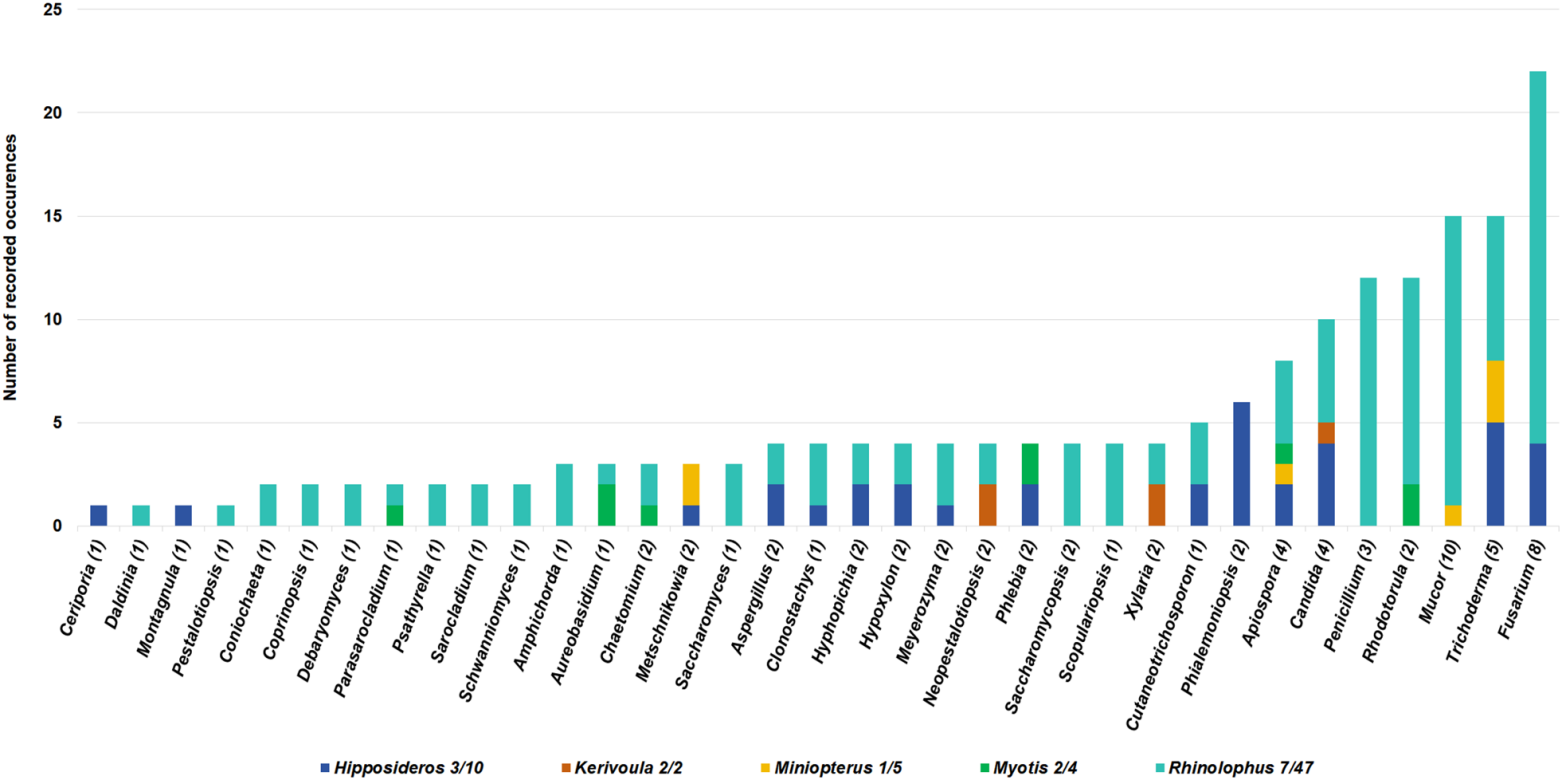
Number of fungal samples cultured per fungal genus, for all genera with at least two separate samples. Numbers shown after the fungal genera indicate the number of fungal species that were found within the genus, numbers after bat names indicate the number of bat species and individuals sampled with culturable fungi. Note that several Hipposiderids had no cultural fungi and are therefore not indicated here

On a generic level, 35 genera of fungi were isolated from bats, of which 24 genera have over two recorded instances, 20 could be pathogenic on both plants and animals, and two were mycoparasitic (Fig. 3, Table 2). Of these, *Fusarium* was the most common, with 22 instances on bats, 16 of which were on *R. malayanus*, four were on *Hipposideros pomona*, and two were on *R. stheno* (Table 2). *Mucor* was the next most common fungal genus, with 17 instances of ten species on seven species of bats, showing a far lower specificity (Table 2). *Trichoderma* followed this with 15 instances of five species (though most were *Trichoderma atroviride* and *T. hipposiderocola*) on five bat species (Table 2). *Penicillium* had 12 instances of three species on four bat species (*Rhinolophus affinis*, *R. rex*, *R. siamensis,* and *R. sinicus*), belonging to Rhinolophids (Table 2). *Rhodotorula* F.C. Harrison also had 12 instances on five bat species, which included only one fungal species, *Rhodotorula mucilaginosa*, and were largely on Rhinolophids (Table 2).

**Fig. 4:**
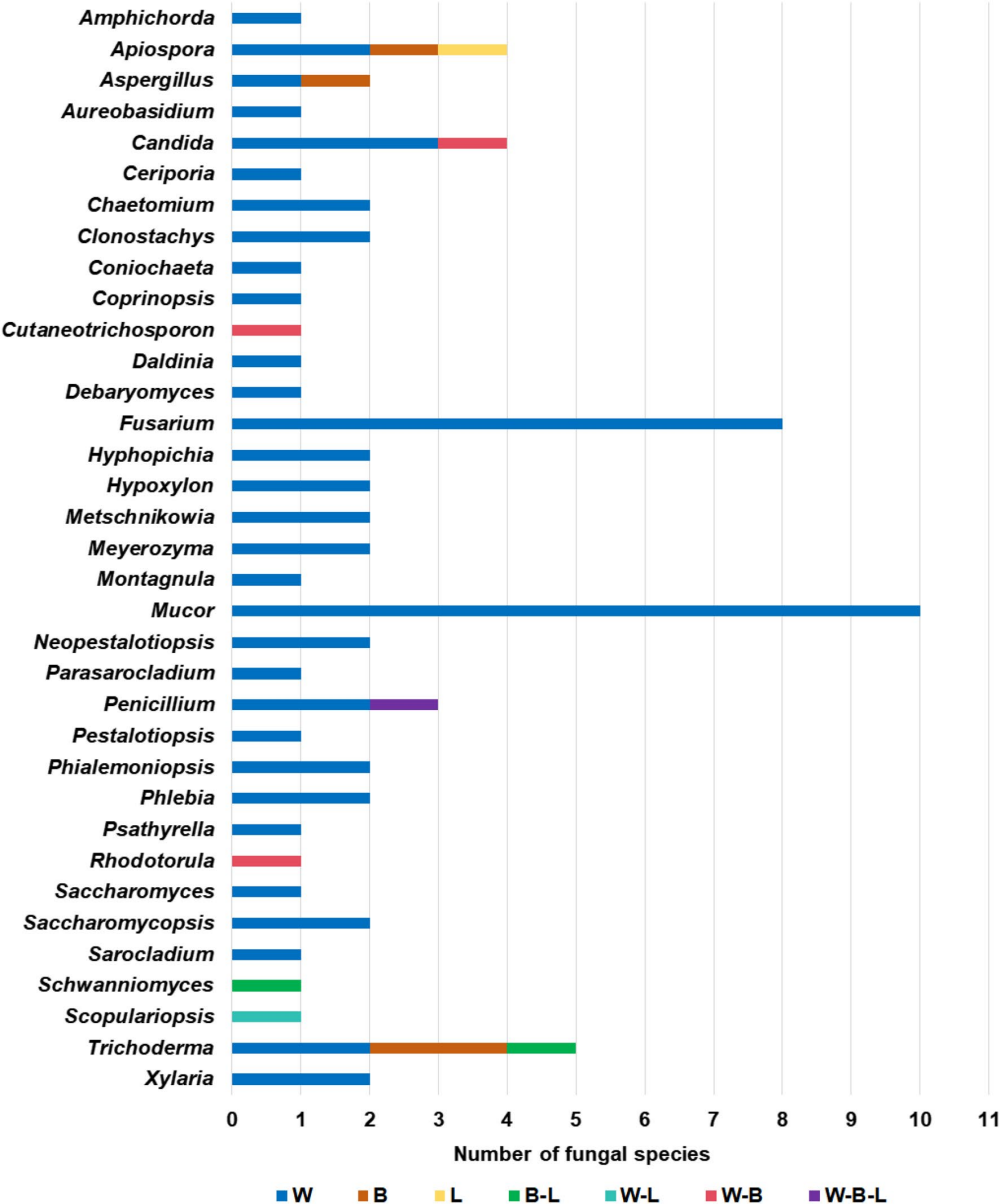
The comparison of fungi genera sampled from bats’ body parts (W-wing, B-body, L-legs)

We found little evidence of host-specificity for most fungi, especially when explored at a generic level. Rhinolophids were the best-sampled group with seven species and 120 individuals, yet only had nine fungal species exclusive to them and present on at least two individuals, and only two (*Penicillium brevicompactum* and *Rhodotorula mucilaginosa*) had multiple incidences. Among all the fungi isolated from bats during this study, *Penicillium brevicompactum* and various *Candida* species were found on multiple bat species. *Trichoderma obovatum* was on three species, but always on the body and sometimes on the legs. Other fungi species and genera were largely restricted to the wings and also showed high species richness among bat individuals (Figs. 3 and 4, Tables 2 and 3). Of the fungal species that could be identified, 35 were known pathogens of either plants or animals, while 13 were exclusive to plants, 22 were exclusive to humans and other animals, two were mycoparasitic fungi, and one was an insect pathogen (Table 2).

### Plant pathogens

*Penicillium brevicompactum* is the most common true pathogen and post-harvest pathogen on numerous crops and plants (Kaitera et al. 2019), and was also present in eight individuals of four Rhinolophoid species. Other plant pathogens were also detected but in lower numbers, including seven *Fusarium* species, viz*., Fusarium annulatum* (causes *Fusarium* rot of cantaloupe melons) (Parra et al. 2022), *F. hipposidericola*, *F. luffae* (leaf blight on loquat, and pokkah boeng of maize) (Parime et al. 2022; Zhang et al. 2023a, b, c), *F. menglaense*, *F. rhinolophicola*, *F. xishuangbannaense*, and *F. yunnanense* (Table 2). In addition, *Apiospora arundinis* (leaf edge spot of peach, leaf blight of tea, and wet root rot of *Pseudostellaria heterophylla* (Thangaraj et al. 2019; Ji et al. 2020; Xiao et al. 2024), *A. marii* (wilt, dieback and tree decline of olive) (Gerin et al. 2020; Farr and Rossman 2022), *Ceriporia lacerata* (white rot fungus) (Suhara et al. 2003; Sui and Yuan 2023), *Chaetomium anastomosans* (diseased root of *Saccharum officinarum)* (Raza et al. 2019), *Hypoxylon investiens* (causes *Hypoxylon* wood rot in tea) (Grand 1985; Otieno 1993), *Penicillium glabrum* (postharvest fruit rot of pomegranate) (Spadaro et al. 2010; Barreto et al. 2011), *Neopestalotiopsis paeoniae-suffruticosae* (diseased branches of *Paeonia suffruticosa*) (Li et al. 2022), *Pestalotiopsis trachicarpicola* (leaf spot of *Eucommia ulmoides*, *Gentiana rhodantha*, *Mangifera indica*, *Podocarpus macrophyllus*, and *Trachycarpus fortunei*, and twig blight of *Pinus bungeana*) (Qi et al. 2021; Zhang et al. 2012, 2021), *Phlebia acerina* (White-rot) (Kumar et al. 2018; Zhang et al. 2023a, b, c), and *P. floridensis* (White-rot) (Magaña-Ortiz et al. 2024) were found on the sampled bats (Table 2).

### Animal pathogens

There are 22 animal pathogens including 19 human pathogens, two bat pathogens, and one other mammalian pathogen. One of the most commonly carried human pathogens found in this study was *Rhodotorula mucilaginosa*, the most common cause of fungemia in humans (Larone 1995; Wirth and Goldani 2012) (Table 2), followed by *Cutaneotrichosporon dermatis* (present on five individuals of four bat species) (Yoo et al. 2022), *Candida parapsilosis* (Trofa et al. 2008), *Mucor variicolumellatus* (Wagner et al. 2020)*, C. saopaulonensis* (fungi infections in premature infant with sepsis) (Ning et al. 2024), and *Scopulariopsis brevicaulis* (Cuenca-Estrella et al. 2003; Woudenberg et al. 2017) (Table 2). *Mucor* was the genus with the highest species richness found on bats*,* including six *Mucor* species, viz*., M. circinelloides* (cutaneous infections of humans), *M. ellipsoideus* (chronic renal failure), *M. irregularis* (an emerging fungal pathogen that causes cutaneous infection of humans and could cause death, and rhinofacial mucormycosis) (Hemashettar et al. 2011; Chander et al. 2015), *M. plumbeus* (able to elicit an immune response in humans by activating the complement system) (Domsch et al. 1995; Kirk 1997; Granja et al. 2010; Boraschi et al. 2020), *M. racemosus* (opportunistic pathogen of immunocompromised individuals such as children, elderly and diseased patients) (Sarbhoy 1966; Inderlied et al. 1985; Alvarez et al. 2011; Gidalishova et al. 2023), and *M. variicolumellatus* (infection of human) (Wagner et al. 2020) (Table 2)*. Apiospora arundinis* (Onychomycosis) and *Chaetomium anastomosans* (eye infections) were both pathogens of humans (Dylag et al. 2017; Vettorato et al. 2020)*.* Two species (*Debaryomyces vindobonensis* and *Hyphopichia burtonii*) were pathogenic on bats (Simpson et al. 2013; Tamayo et al. 2021), were found on two bats from two different families, suggesting these fungi may be relative generalists within bats. In addition, *P. coprophilum* was a pathogen on mosquitoes (Costa et al. 1998).

### Non-pathogenic fungi

In addition to plant and animal, fungal pathogens, we isolated 39 non-pathogenic fungal species from bats, including 11 new species associated with bats in Liu et al. (2023), and 28 other species are important are saprobes or endophytes. Some of these species have important roles in ecosystems and agricultural production. *Metschnikowia koreensis*, one of the nectar-specialized yeasts of genus *Metschnikowia* has been shown to influence pollination by altering the strength of plant-pollinator interactions through modification of the chemical properties of nectar (Grigoriev et al. 2014; Canché-Collí et al. 2021). *Parasarocladium gamsii* can enhance plant growth and modulate plant genes to mitigate soil stress in plants (Furtado et al. 2021). *Saccharomycopsis crataegensis* is a predacious yeast, which can used to control postharvest decay of oranges caused by *Penicillium digitatum* (Pimenta et al. 2010), and reduced concentration of aflatoxins in peanuts caused by *Aspergillus parasiticus* (Prado et al. 2008). *Saccharomycopsis fibuligera* was widely found in all types of fermentation starters, and used to produce ethanol from starch (Chi et al. 2009; Xie et al. 2021). *Sarocladium zeae* is a systemic endophyte of wheat and corn, can be used as an effective biocontrol agent *Fusarium* head blight (Kemp et al. 2020; Liu et al. 2022; Noel et al. 2022). *Schwanniomyces polymorphus* may can help ants to more efficiently assimilate nutrients when fed nutrient-deficient diets (Mankowski et al. 2021). *Xylaria curta* has a potential value in the clinical field with the activity of xylarichalasin A produced against cancer, and resistance reversal activity against fluconazole-resistant *Candida albicans* (Wang et al. 2019; Becker and Stadler 2021).

### Host preference and host-specificity

**Fig. 5:**
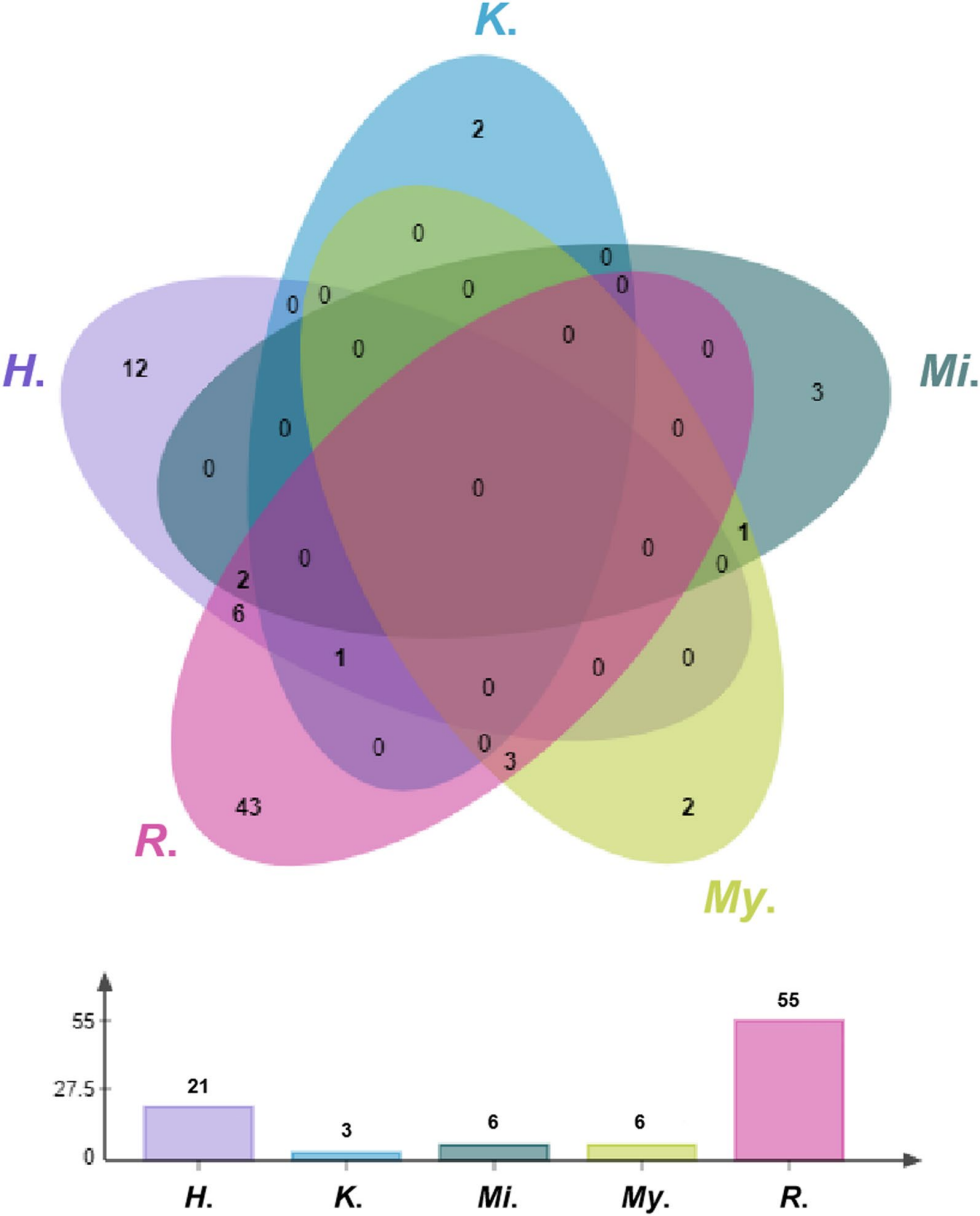
Number of culturable fungal species from different bat genera (*H*.: *Hipposideros*, *K*.: *Kerivoula*, *Mi*.: *Miniopterus*, *My*.: *Myotis*, and *R*.: Rhinolophus)

In total, 75 culturable fungal species were isolated from five bat genera, including 43 fungal species only isolated from *Rhinolophus*, 12 species only isolated from *Hipposideros*, three species only isolated from *Miniopterus*, and two species only isolated from *Myotis* and *Kerivoula,* 11 species from two genera, and two species from three genera (Figs. 1 and 5, Table 2). Almost all culturable fungi were isolated from insectivorous bats, and only one species (*Apiospora arundinis*) was also from a fish-eating bat (*Myotis pilosus*) (Table 2). According to our data, there is little host preference or host-specificity between the fungal and bat taxa, though further data is needed (Table 2).

### Determinants of fungi present

Only fungi that could be cultured are represented in our study; thus, unculturable and probably slow-growing fungi remain unrepresented in our dataset. That said, the incidence of fungi found on the bats varied by bat group, even for those with larger sample sizes. For example, Rhinolophids typically had a higher incidence than Hipposiderids, particularly large Hipposiderids; for example, among the captured large aggressive Hipposiderids, such as *H. larvatus*, only 36.36% hosted culturable fungi. The smallest Hipposiderids, such as *H. cineraceus* and *Aselliscus stoliczkanus* (though only represented by a single individual), had no culturable fungi. The number of fungal species on an individual bat was highest on *Hipposideros larvatus*, *Rhinolophus malayanus*, and *R. stheno*, with five species on each. The average (mean) number of fungal species on an individual bat was highest on *Hipposideros larvatus* at three species, followed by *H. armiger* at 2.75 species, *Rhinolophus affinis* at 2.67 species, and *Kerivoula papillosa*, *Miniopterus schreibersii*, *Myotis laniger* and *R. rex* at two. The results for *Rhinolophus malayanus* are striking because despite having 20 individuals for which fungi could be cultured, the average was only 1.65 fungal species per individual, and 64.52% of individuals had culturable fungi. Conversely, over half of *Rhinolophus stheno* (58.82%) had no culturable fungi, and further data is needed to understand these varying levels of occurrence.

However, understanding how incidence varies per species should also be in the context of factors such as site. On average, the number of fungal species per bat was highest in both the limestone forest in Xishuangbanna and the cave at Pubei, with an average (mean) of 1.9 each, followed by the rainforest at 1.6 and the cave at Xishan at 1 (Table 1). At a site level, most species that are found at multiple sites are found in both the limestone and the rainforest, but abundance and diversity are highest in the limestone rainforest; for many species, average fungal species richness was higher in the limestone forest, and possibly highest in Pubei (Fig S3), though lack of shared species between many sites makes such comparisons challenging.

## Discussion

Fungi have become increasingly recognized as important pathogens in various systems, including increasing numbers of cankers, crop pathogens, major epidemics such as the chytrid fungus in anurans (frogs and toads), and white-nose syndrome in bats. White-nose syndrome is estimated to have killed at least seven million bats in the US, underscoring the importance of understanding these pathogens, especially with climate change. Crop fungi are estimated to cause approximately 30% of crop losses annually, while these losses in major crops are estimated to be enough to feed 8.5 − 61.2% of the world’s population (Fisher et al. 2012), in addition to widespread cankers and blights in tree crops. Yet, how these fungi are distributed across the landscape is relatively unknown. Here, we show the capacity of bats to act as vectors for these fungi, moving them across the landscape and acting as a possible conduit between natural and agricultural areas. It is important to note that whilst we could culture 75 fungal species on bats, many species may not be culturable in media, and thus other fungi (including pathogens and non-pathogens) may also be present and calls for further work to identify and explore these unculturable species.

### Major pathogenic fungi associated with bats

In total, 34 of the most common fungal genera found on bats in this study were found to be capable of infecting humans, other animals, and plant hosts, including 11 fungal species infecting plants, 20 which infect animals, one mycoparasitic fungi, and two infect both plants and animals (Table 2). Most of these were not restricted to a single bat genus, showing that many are potentially generalists. These also included several pathogens affecting crops grown in the area, including grapes, tea, maize, and other cash crops, and thus bats may act as a significant vector for fungal pathogens in these groups. Previous studies have found 50 species of pathogens on bats and in bat-associated environments (Karunarathna et al. 2023; Liu et al. 2023), and our current study further adds to this list. These pathogens are easily transported across different land use systems frequented by bats, transmitting pathogens between natural landscapes and agricultural environments (Karunarathna et al. 2023). For example, the plant pathogens *Apiospora arundinis*, *Fusarium annulatum*, *F. luffae*, *Penicillium brevicompactum*, *P. glabrum*, and *Hypoxylon investiens* were some of the most common taxa in our study and can devastate certain crops. Aflatoxin contamination of maize crops from fungal growth is estimated to cost somewhere between $52.1 million to $1.68 billion annually in the US alone (Mitchell et al. 2016); thus, understanding routes of contamination is critical in developing effective ways to mitigate the spread (Khlangwiset and Wu 2010) since bats transport *Aspergillus* and *Penicillium* species. Likewise, *Fusarium* also costs billions a year in the US (Wilson et al. 2018)*.* The impact of fungal pathogens on agriculture will only intensify as human-disturbed landscapes multiply due to the increasing need for bats to distribute through fragmented landscapes to forage effectively and navigate between remaining habitat patches. Evidence shows that bats host and transport plant pathogens across their natural ranges (Karunarathna et al. 2020), with serious implications for global food security, and some of the fungi found most frequently on bats in this study are already known to cause major economic losses in crops.

Our analysis shows that bats irrefutably harbour and transport fungal pathogens which impact both plants and animals. Emerging infectious fungal diseases from bats and bat habitats could be potential sources for future infections in human populations (Karunarathna et al. 2023). Currently, the spread of plant pathogens represents the most pressing threat (Fisher et al. 2012) from bat-associated fungi, which should be considered in future landscape management strategies, especially with the expansion of agricultural lands into natural habitats, fragmented forest environments, and increased probability of bats acting as vectors. These all represent considerable threats to human health. Furthermore, as some of these species will roost or temporarily roost in buildings, including agricultural storage areas, their ability to spread fungi within stores (including grain stores) should not be overlooked.

### The role of bats as fungal vectors

Bats are vectors of both pathogenic and non-pathogenic fungi, and understanding how bats act as potential vectors is crucial to mitigating possible risks. However, it is important to note that their role in spreading fungi is still likely relatively low, especially as the bats with most fungi here were forest dependent Rhinolophid species, which are unlikely to pass through agricultural systems, whilst these species also provide key services such as pest control. Here, we show that most bats carry multiple fungal species, and many may become human or crop pathogens. The incidence does vary, however, for example, 77.14% of fungal genera were present on Rhinolophid species, relative to 42.86% on Hipposiderids, 17.14% on *Myotis,* and only 11.43% and 8.57% on *Miniopterus* and *Kerivoula* respectively. Interestingly, the body part on which the fungi grow also varies between bat species, with Rhinolophids showing the highest incidence and diversity of fungi on their wings, whereas Hipposiderids and *Myotis* having a much lower incidence on wings (noted when multiple body parts were examined). Within the Rhinolophids, *R. rex* was the exception, with a higher diversity of fungi on the body, possibly due to the greater fur length (Rhinolophids are known as woolly bats, but typically only larger species have longer fur (*R. rex* was the largest Rhinolophid sampled here)*.* Given that bats inhabit a thermally stable environment during the day and forage whilst the climate is cooler and more stable, they have the ability to provide a thermally stable environment, which may be optimal for many fungi (Liu et al. 2023). However, our results show that species and site level differences dominate, and thus, understanding the ability of bats to act as vectors will require further work, which unpicks the impacts of species-specific traits, demography, seasonal changes, and landscape structure.

Due to the small sample size, and short sampling period, which does not span across the year, we could not explore annual trends or how they may vary by sex. These factors are likely to alter the observable patterns in fungal growth as they impact the possibility of spreading fungi between individuals. These impacts are also expected to vary in species with high levels of aggression, especially if this varies by sex, such as large Hipposiderids, where aggression, particularly in males in larger species, increases the distance between individuals in a roost (Zhang et al. 2023a, b, c). We also found that some sites had a higher individual richness of fungi than others, even within a species, for example, the limestone forest and the Pubei site, which may relate in part to the agricultural matrix these sites are situated in, but different species composition at each site makes it challenging to disentangle these factors. However it is important to note that species studied here are generally forest dwelling species, and the loss of forest habitats will increase the probability that bats will need to tranverse or forage over agricultural habitats, potentially both increasing the risk of increasing exposure to fungal pathogens, as well as pesticides which may adversely affect bats.

### Bat species traits and propensity to act as fungal vectors

The probability of being exposed to fungi is a function of either environmental exposure or exposure from another bat (Liu et al. 2023). From either of these sources, exposure may be airborne (or within water droplets) or through direct contact with infected surfaces. The differences in fungi cultured on bats of the same species, at the same time, means that at least during non-hibernation conditions, relatively few fungi may be passed among individuals within a roost. However, in species where we did find more individuals hosting the same fungi, the bats tended to be small, cave-roosting Rhinolophids. In at least some bat groups, there is a relationship between body size and aggression, and in such species, individuals will always maintain a certain distance from each other when roosting. Understanding species-specific roosting traits is critical for understanding potential fungal exposure, as different species can have different roosting preferences (Rosli et al. 2018). In *Hipposideros*, large species tend to be very aggressive (Sun et al. 2018, 2021). Within our study, we caught two large Hipposiderid species, and in both cases, they had relatively lower percentages of culturable fungi (25% n: 4 *H. armiger*, 36.36% n: 11 *H. larvatus*), which is likely related to significant distances between individuals during roosting (Selvanayagam and Marimuthu 1984). For medium Hipposiderids, *H. pomona* had higher percentages (57.14%, n: 7) of culturable fungi, whereas, for the smallest Hipposiderids, neither *H. cineraceus* nor *Aselliscus stoliczkanus* had any fungi. In the case of *Aselliscus stoliczkanus* in this area, most individuals roost singularly in gaps between stalactites or bell holes of caves, and in other regions, most individuals roost with a significant distance between them, typically becoming torpid during the day even in prevailing warm conditions (Hughes et al. 2023). Meanwhile, *H. cineraceus* shows very low local abundance. Conversely, small Rhinolophids typically roost very close to each other, except for *R. stheno*, which had a much higher incidence and fungal diversity than most Hipposiderids, with the maximum of fungal species in a Rhinolophid higher than any other species examined with individuals hosting 3–5 species. It should be noted that these studies were conducted in a tropical and subtropical area during the warmer parts of the year, and the level of individual similarity in terms of species hosted by different bats would likely have increased during hibernation in the temperate Northern parts of Yunnan, where certain species will cluster together to minimize energy loss and maximize heat-retention during hibernation (Martínková et al. 2020), also providing the ability to transfer fungi between individuals.

Temperature is also important, whilst caves are thermally stable, different bats have different thermal regulation abilities and habits, with some individuals, such as *A. stoliczkanus*, regularly becoming torpid during the day (Geiser 2004) and capable of showing similar temperatures to the background environment (Bartonička et al. 2017). Temperature is a critical factor, as minimum temperatures during winter have been shown to relate to the spread of white-nose syndrome in North America, as certain fungi can only survive above a minimum temperature (Martínková et al. 2018; Turbill and Welbergen 2020). Understanding temperature variation in the landscape, as well as species-specific thermal profiles, may alter what fungi they are likely to host.

Bat wings also determine what fungi may be present by providing the fungi with a substrate to grow on and altering species’ behaviour (as wing dimensions relate to habitat use) and, therefore, exposure. Vascularization patterns and wing structure vary considerably between species (Cheney et al. 2017), which may influence the growth of any fungi showing any degree of pathogenicity on the bat. These factors are likely to have at least some impact, as the number of fungi on bat wings and the number of individuals who hosted fungi on their wings but no other body parts suggests that the wings provide ideal conditions for fungi to grow on. Furthermore, bat wing structure (wing loading, aspect-ratio) are largely a product of habitat density, with shorter, broader wings related to densely cluttered forested landscapes and longer, thinner wings associated with open areas. There is also a high degree of phylogenetic conservatism in the bat wing structure, with the majority of Rhinolophids largely restricted to forested areas (Wang et al. 2010), whereas Hipposiderids (particularly larger species) regularly use more open areas (Lee et al. 2012). This use of habitat influences both exposure to various fungal pathogens and, thereby, the ability to spread such pathogens across the landscape. Species such as *H. larvatus* have been shown to even carry viral pathogens such as porcine diarrhoea virus (Zhou et al. 2021), possibly as a consequence of foraging or even roosting in agricultural fields and buildings. Conversely, *Rhinolophus* is more likely to forage in tree and vine crops, where they are known to contribute to pest control (Baroja et al. 2019).

The usage of different parts of the landscape based on species and genera-specific traits alters the capacity of various species to transmit fungi among agricultural, natural, and cave systems. These factors also vary by season, as species change habitat use based on the reproductive phase, in addition to migration and hibernation in temperate landscapes (Kunz et al. 2003). Whilst large Hipposiderids are known to migrate large distances, further research is needed to explore these patterns (Vaughan 1977; Crichton and Krutzsch 2000; de la Pena-Cuellar and Benitez-Malvido 2021; Meng et al. 2021). All these factors alter the ability of fungi to spread between either individuals or across the landscape, in some cases over extended distances through migration. Furthermore, some species may use different roosts during the day and temporarily through the night whilst foraging, again leading to differential exposure in these systems (which may, for example, include buildings for species adapted for foraging in open areas) (Crichton and Krutzsch 2000; Kunz et al. 2003; Lacki et al. 2007).

### Implications for fungal pathogen spread for different taxa

As we show, different individuals and species of bats can host very different fungal populations, and local landscapes can have significant implications for the spread of fungi. Loss of native forest sites will increase the need for bats to forage in agricultural areas (Kalda et al. 2015; Blary et al. 2021) or commute between natural areas. In these instances, the probability of exposure to crop pathogens increases, even for clutter-dependent forest species, and fragmented landscapes only increase this exposure. Furthermore, whilst bats contribute significantly to pest control, the use of insecticides not only decreases insect populations but forces bats to forage over larger areas to obtain enough nutrition. Greater foraging increases exposure to both fungal pathogens and agrochemicals, which could impact immune function, as has been shown in previous studies (Oliveira et al. 2021), especially as much of this is through the skin (EFSA et al. 2019) and could change susceptibility to fungal infection.

In addition, loss of roost sites may force animals to distribute into suboptimal roosts where other species are present or into buildings (Frick et al. 2020; Crawford and O'keefe 2023), which may increase exposure to fungi within other systems, in addition to increasing the potential for spread between individuals. With a 5.7% loss of karst per year within regions like Southeast Asia (Hughes 2017), and no mitigation measures for roosts displaced during construction in much of the world, roost-site disturbance and loss present a significant risk of impacting patterns of fungal spread. This also highlights the need for better seasonal data, as physiological status may change susceptibility, and movement across the landscape (especially if disturbed) will alter exposure. Periods such as hibernation, in particular, require further study, as both white-nose syndrome and observations of potentially pathogenic fungi in China have both been evident during hibernation when species down-regulate most biological processes.

### Need for OneHealth approaches to minimize the risk of fungal spread

Bats act as vectors for significant numbers of fungi. Their ability to act as vectors to crops or animals is very much a product of how landscapes are managed and the increasing reliance of bats on agricultural landscapes for foraging as natural habitats continue to be lost. Whilst bats contribute millions of dollars annually through pest control and pollination services (Boyles et al. 2011; Riccucci and Lanza 2014), exposure to fungi whilst foraging has a potential downside to the ecosystem services bats provide, especially as concurrent exposure to pesticides may alter susceptibility to various pathogens. Managing such a balance is challenging but involves ensuring that bats have adequate access to natural areas to reduce the exchange between natural and agricultural areas (and stable cave environments, which may host fungal populations over extended periods). Optimizing pest control whilst minimizing risks of fungal pathogen spread may involve managed populations of bats within agricultural landscapes, such as the Florida bat houses (https://www.floridamuseum.ufl.edu/bats/) estimated to host over 300,000 individuals. Such approaches ensure the advantages provided by bats whilst minimizing the need for pesticides and thereby reducing both costs and negative impacts of chemicals in the environment. Such bat houses could also be scaled to the size of the agricultural area, and for forest-based ecosystems, having a buffer of shorter vegetation or crops may reduce the use by clutter-adapted species (which hosted more diverse fungi within this study). Such an approach may make areas more attractive to bat species that rely on a hawking foraging approach (appropriate for open areas) rather than a gleaning approach, which would also reduce direct contact between bats and crops. Whilst some of these species travel huge distances, their high flight paths are likely to reduce exposure to fungal pathogens (Horn and Kunz 2008). Bats have also been shown to suppress pest-associated fungal growth and mycotoxins in corn (Maine and Boyles 2015), but further studies are needed to verify the extent of this. Additionally, to enable bats to commute through landscapes, buffer strips to hedgerows may reduce exposure to both pathogens and crops and may follow existing legislation such as the EU habitats directive (Mehtälä and Vuorisalo 2007). Furthermore, as many bats rely on caves, humans visiting these species (such as speleologists) must take care to ensure all equipment is washed and dried thoroughly before any cave visit to avoid the movement of fungal pathogens between caves, and between caves and other parts of the landscape.

Our work provides an initial insight into the role of bats as fungal vectors across landscapes, ultimately, a OneHealth approach is needed to manage landscapes and minimize risks. This means ensuring sufficient intact habitat exists to meet species needs, including foraging and roost sites. Within buildings and bridges, effective mitigation should be set up upon development to prevent novel community aggregations (Sutherland et al. 2020). Minimizing exposure to chemicals and managing agricultural landscapes are also critical to prevent bats from acting as vectors of fungal pathogens within these landscapes. Furthermore, for cave bats in particular, given the popularity of tourism, hygiene, and biosafety standards should be maintained before and after entering roost sites to prevent the spread of possible fungal pathogens. In addition, mining activities should proceed with stricter environmental oversight, as disturbance and resettlement of bats could spread potential pathogens. Ultimately, mitigating risk means minimizing the interface between systems that bats may otherwise transport fungi between and maintaining healthy native populations, which requires a more holistic approach to managing natural and agroecosystems. An interdisciplinary effort will be needed to develop strategies to ameliorate and prevent the emergence or spread of bat-associated fungal diseases. Zoologists, mycologists, speleologists, and medical scientists must collaborate to bolster our understanding of the complex interplay between bats, their habitats, and the fungal species in these systems.

## Conclusions

Bats are known vectors of various pathogens, but their role of potential dispersers of fungi has not previously been examined. Yet, following the major mortality of bats associated with White Nose syndrome (*Pseudogymnoascus destructans*) understanding interactions between bats and fungi, and the potential for further fungi to pose potential risks to bats is clearly needed. Furthermore, given the ability of bats to traverse the landscape, and roost in a thermally stable environment, the potential ability of bats to disperse fungi across the environment warrants further study. Within our study we isolated 75 culturable fungal species, of which 36 were pathogenic and 39 non-pathogenic or unknown. A total of 68 bats were found to have culturable fungi, 96 had none, with 48% of fungal species (36 species) representing known pathogens of plants, animals, humans, mushrooms and insects, and 52% (39 species) representing known non-pathogenic fungi. This included a wide diversity of fungi (77% (58 species) Ascomycota, 9% (seven species) Basidiomycota, and 13% (10 species) Mucoromycota). Furthermore, we found some evidence for specificity both of fungal species on specific bat species (though little at a genus level), and on particular tissues on bats, with a disproportionally high number of fungal species found on the wings. As bats may move between habitats, including crops, or livestock enclosures, they clearly show the ability to transport fungi across the landscape, posing a risk of transferring fungal pathogens. Further loss and degradation of habitats may increase the need of bats to move across the landscape, increasing exposure, and the potential to transfer fungi across landscapes. Mitigating this risk will require better measures to manage landscape, and reduce the need of bats to traverse highly agricultural and other developed landscapes, and therefore calls for enhanced measures to protect intact habitats and maximise connectivity within agricultural systems.

## Data Availability

All data generated or analyzed during this study are included in this published article.

## Declarations

### Adherence to national and international regulations

All regulations were followed in accordance with national and international policies.
